# Conditions Affecting the Release of Heavy and Rare Earth Metals from the Mine Tailings Kola Subarctic

**DOI:** 10.3390/toxics9070163

**Published:** 2021-07-09

**Authors:** Eugenia Krasavtseva, Victoria Maksimova, Dmitry Makarov

**Affiliations:** 1 Laboratory of Nature-Inspired Technologies and Environmental Safety of the Arctic, Kola Science Centre, Russian Academy of Sciences, 184209 Apatity, Russia; v.maksimova@ksc.ru; 2 Institute of North Industrial Ecology Problems, Kola Science Centre, Russian Academy of Sciences, 184209 Apatity, Russia; d.makarov@ksc.ru

**Keywords:** heavy metal pollution, risk assessment, availability, rare earth elements, climate

## Abstract

In the Kola Subarctic, a mining industry has developed, which is a source of environmental pollution with heavy metals. The objects of study were the tailings of three large mining enterprises in the region: apatite-nepheline, complex and loparite ores. The geotechnical characteristics were studied, and the granulometric composition of the samples was established. The main minerals that make up the material of ore dressing tailings have been determined. Using inductively coupled plasma mass spectrometry, the content of trace elements, in particular heavy metals and rare earth elements, has been established. The enrichment factor, the geoaccumulation indexes, the potential ecological risk index factor and the potential environmental hazard index have been calculated. Priority pollutants characteristics for specific objects have been identified. It is noted that the finely dispersed material of the tailings of loparite and complex ores is 1.5–3 times enriched in heavy and rare earth metals in comparison with the total material of the tailings. In laboratory conditions, experiments were carried out to simulate the process of interaction of dust particles with soil solutions containing different amounts of dissolved organic matter and at average seasonal temperatures. It was found that a decrease in the pH of the solution and an increase in the amount of organic carbon and temperature lead to the mobilization of heavy and rare earth metals from the tailings.

## 1. Introduction

The active development of the mining industry in the Arctic regions leads to an increase in the level of environmental risks for the environment. To protect the unique natural environment of the Barents Euro-Arctic Region, a wide range of research and international initiatives are currently being carried out on various topics, including climate change, biodiversity and natural habitats conservation, elimination of chronic stress for ecosystems, study and reduction of pollutant emissions substances and the introduction of green production technologies.

The study of various aspects of the negative impact of mining waste on environmental components in the regions belonging to the Arctic zone was carried out in many articles, including [1,2,3,4,5]. The main method of disposal of mining and enrichment wastes around the world is still filling surface tailing dumps [6], which are a source of significant environmental and social risk [7].

Considering tailing dumps as sources of long-term impact on adjacent environments requires not only an assessment of the content of individual pollutants in environmental components in comparison with the standards established in environmental documents but also special attention to the problems of toxicity and the level of accumulation of pollutants in organisms, interaction with active components of the natural environment, such as precipitation and soil solutions during penetration into deeper soil horizons and during overgrowing of dumps and tailings with primary successions.

The Murmansk region is one of the most urbanized and industrially developed regions of the Arctic zone of the Russian Federation. Deposits of apatite-nepheline, copper-nickel, magnetite, loparite ores, niobium and tantalum, etc., are being developed in the region, increasing in comparison with 2018 by 13% and amounted to 259.5 million tons [8]. This is 99.8% of the total generation of all types of production and consumption waste in the Murmansk region.

Tailings dumps have a long-term multifactorial impact on the environment. In the thickness of the tailings, the processes of chemical interaction of minerals with atmospheric and filtered circulating waters continuously occur. The crushed material has a high specific surface area and interacts more actively with washing solutions. The presence of a fine fraction in tailings often leads to the dusting of tailings during the snowless period and the transfer of pollutants over considerable distances. In particular, this is typical for the ANOF-2 tailing dump of Apatit JSC in the Murmansk region [9]. Active migration of pollutants leads to landscape degradation, deterioration of the quality of soils and water bodies and has a negative impact on the quality of life and health of the population of the Murmansk region living in close proximity to mining enterprises. Soluble metal compounds are easily included in the biogeochemical cycles of elements and ultimately affect the state of ecosystems, biota and the quality of life of the population. The airborne transport of dust particles from tailings leads to a decrease in air quality and a change in the chemical composition of aerosols [10,11]. The effect of particles with a size of PM10 and less is currently the object of close attention of researchers in the medical and biological direction, and now, the already recognized consequences of long-term exposure to particles on the human body are respiratory and cardiovascular diseases, severe intestinal disorders, keratosis, skin cancer and others [12,13,14,15,16]. Plants growing in areas near mining enterprises are contaminated both due to dusting and due to the absorption of pollutants from the soil solution [17].

The mobility of pollutants entering the environment from tailings is influenced by external environmental conditions, including the pH level of the environment, the chemical composition of the soil solution and the presence of dissolved organic matter. In [18], a general similarity in the behavior of heavy metals (HM) and rare earth elements (REE) in the soil solution was noted. At the same time, metal cations interacting with dissolved organic matter are involved in a wide range of physical, chemical and biochemical processes, one of which is the formation of organomineral compounds [19]. A consequence of a wide variety of interaction options is the possibility of both increasing the intensity of migration of pollutants, and vice versa, their fixation in the soil, depending on the specific geochemical setting.

The influence of climate on changes in soil properties and, as a consequence, on the mobility of metals is presented in [20,21]. Dissolved forms of metals have a greater effect on the biota state than the total content of the pollutant [22,23]. Understanding the scale and ways of spreading pollution from tailing dumps, the influence of climatic and hydrochemical factors is important for controlling the level of environmental pollution and developing methods to reduce it. Such studies are of particular value in regions with special climatic and geographic conditions and high anthropogenic pressure.

The purpose of this work is to assess tailing dumps as a source of soil pollution with heavy and rare earth metals in the Kola Subarctic and to determine the influence of environmental factors on the mobilization of pollutants.

## 2. Objects and Methods

### 2.1. Geography and Climatic Characteristics of the Study Area

The Murmansk region is located in the north-west of the European part of Russia, with borders in the south-west with the Republic of Karelia (Russia) and in the west and south-west with Finland and Norway. Almost the entire territory of the Murmansk region lies north of the Arctic Circle, washed in the north by the non-freezing Barents Sea, in the east and southeast by the freezing White Sea. Climate—arctic-temperate, maritime, softened by the influence of the Gulf Stream branch, belongs to the Dfc class according to the Köppen–Geiger climate classification (subarctic climate; coldest month with an average temperature below 0 °C or −3 °C and with an average temperature of 1–3 months above 10 °C). The relief is mainly represented by two physical and geographical zones—tundra and taiga. The average air temperature in the coldest months (January–February) ranges from −8 °C in the north of the region (the influence of a warm current) to −12 … −15 °C in the central regions. In summer, respectively, +8 °C and + 14 … + 15 °C.

With a fairly small total area (0.85% of Russia’s area [24]), the region is not only strategically important due to its geopolitical position, but it is also one of the most economically developed regions of the Arctic. On the territory of the Kola Peninsula, there are about 60 large deposits of mineral raw materials, the most valuable of which are copper-nickel, apatite-nepheline, iron and rare earth ores. The region produces 100% apatite, nepheline and baddeleyite concentrates, 10% iron ore concentrate, and 7% refined copper, and it is a major copper producer in Russia. Large centers of the mining and metallurgical industries located in the Murmansk region have a negative impact on the environment.

**Figure 1:**
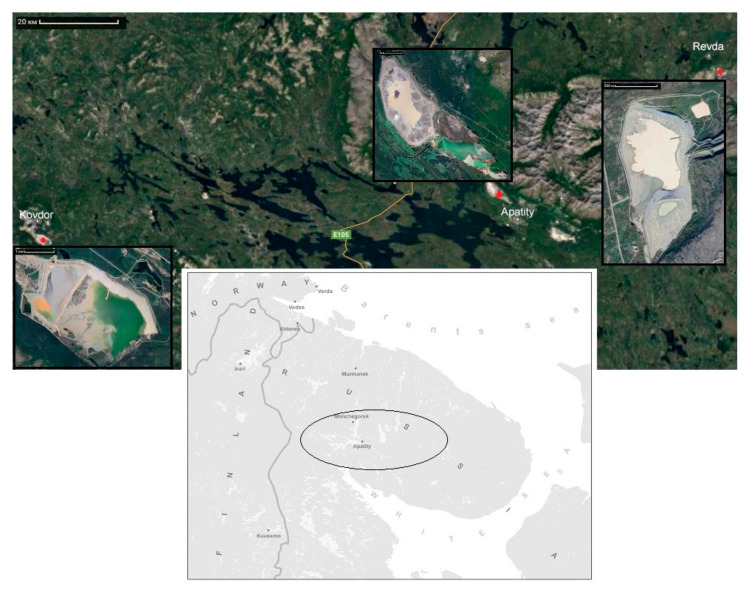
A schematic map of the study area.

The objects of study were the tailings of the concentration of loparite, apatite-nepheline and complex ores (Figure 1).

The loparite ore dressing tails were collected at the tailing dump of an enterprise developing a deposit of niobium, tantalum and rare earth elements of the cerium group. The commercial product obtained at the enterprise—loparite concentrate—is a complex raw material for further production: tantalum, niobium, cerium group of rare earth and titanium [25].

The apatite-nepheline ore dressing tails were taken at the tailing dump of an enterprise that develops the Khibiny deposits of apatite-nepheline ores, produces them and produces them. Apatite concentrate is the main product of the enterprise.

The complex ore dressing tails were collected at the tailing dump of an enterprise that carries out complex processing of mineral raw materials using low-waste technologies and the production of three commercial concentrates: iron ore, apatite and baddeleyite [26].

### 2.2. Tail Physico-Chemical Analyses

Samples of the tailings were taken by the cutting ring method (ring diameter 70.0 mm according to [27]), used for cohesive (easily cut out) and loose soils.

In laboratory conditions, sieve analysis and engineering-geological characteristics of the selected material were carried out in accordance with [27]: moisture, density and porosity. The particle size distribution was determined using a sieve analyzer model AS-200U. We used sieves with different mesh sizes, mm: 1, 0.5, 0.25, 0.1, etc. The analysis was carried out in accordance with [28]. The work also used data obtained by colleagues from the Mining Institute of the Federal Research Center of the Kola Science Centre Russian Academy of Science [29].

Mineralogical and silicate analyzes of the selected samples were carried out in accredited laboratories of OJSC “Kola Geological Information and Laboratory Center.”

The qualitative composition of the samples was controlled by powder X-ray diffraction on a DRON-2.0 X-ray diffractometer, CuKα radiation. The PDF2 database was used to identify the phases [30].

To measure the total content of elements, a 200 mg sample of the ground was subjected to open acid decomposition with a mixture of HNO_3_, HF, HCl in glass-carbon crucibles. After decomposition, the solutions were transferred into polypropylene 50- or 100-mL tubes, which were filled to the mark with a 2% HNO_3_ solution. A 2% HNO_3_ solution was also used to dilute the solutions.

Analysis was performed using an ELAN 9000 DRC-e inductively coupled plasma mass spectrometer (by Perkin Elmer, Waltham, MA, USA). To calibrate the instrument, we used the standard solutions ICP-MS Calibration Standard IV-STOCK-21 and IV-STOCK-29 (by Inorganic Ventures, Christiansburg, VA, USA) with a mass concentration of the measured elements of 10 mg/dm^3^. The measurement error did not exceed 0.5% at *p* = 0.95.

### 2.3. Heavy Metal Pollution and Risk Assessment

The hazard assessment of the studied tailings was carried out on the basis of the calculated coefficients and indices: the enrichment factor, geoaccumulation index, potential ecological risk index factor and potential ecological hazard index.

The enrichment factor EF_i_ is the enrichment factor for pollutants, metals.

EF𝑖 = 𝐶𝑖⁄𝐶𝑏𝑖, where C_i_ is the metal concentration for each investigated area; C_bi_ is the background concentration of the metal, in our case, the clarkes of the element’s content in the C horizon according to [31]. Due to the possible difficulties of finding the primary source, Table 1 shows the clark content for the elements under consideration.

The EF values were interpreted as suggested [32,33], which distinguish 7 EF classes in Table 2.

Another commonly used tool to evaluate trace metal pollution is the geoaccumulation index (Igeo), which determines and defines metal contamination in sediments [34]. Igeo enables an assessment of environmental contamination by comparing differences between current and pre-industrial concentrations of pollutants [34,35,36].

I_geo_ = log_2_ (C_n_/1.5B_n_), (1)

where C_n_ is the measured total concentration of HM determined in a soil (mg / kg), and B_n_ is the geochemical background value of the elements, in our case, the clarkes of the element’s content in the C horizon according to [31]. The constant in the equation—1.5—is used to analyze natural fluctuations in the environment and very small anthropogenic impacts [36]. I_geo_ consists of 7 classes as shown in Table 2.

The potential ecological risk index factor and potential ecological hazard index are also applied. These indexes can comprehensively evaluate concentration effects, toxicities and ecological sensitivities of HM [37,38,39]. The potential ecological hazard index was formulated by Hakanson (1980), which integrated the concentration of heavy metals with ecological effect, environmental effect and toxicology, and was used to assess the heavy metals pollution ecological hazard for sedimentology [40]. According to this method, the potential ecological risk index factor (E_ir_) of an individual element and the potential ecological hazard index (RI) of a multi-element element can be calculated using the following equations:

E_ir_ = T_i_ × C_n_/B_n_ (2)

where T_i_ is a factor of the toxic reaction of an individual toxic element. The established T_i_ values are 1 for Zn, 2 for Cr, 5 for Ni, Cu and Pb, 10 for As and 30 for Cd [39].

RI = ∑ E_ir_ (3)

According to Hakanson (1980), the following classification is proposed for the E_r_ and RI values presented in Table 3.

### 2.4. Laboratory Experiments

In laboratory conditions, we simulated the interaction of a fine fraction of tailings with model solutions that simulate soil waters. To assess the influence of various factors (pH, amount of dissolved organic matter, temperature), the experiment was carried out in several stages.

In a series that determines the effect of the amount of organic matter, we used model solutions containing about 50 and 100 mg/L of dissolved organic carbon (total organic carbon (TOC)). As a source of water-soluble organic matter, we used high-type milled peat (State Standard 52067-2003). The aqueous extract was prepared at an S:L ratio of 1:1.5 (1) and 1:3 (2) for 24 h, the resulting solution was filtered through a double paper filter “blue ribbon”.

In the series that determines the effect of temperature, a water extract of soil sampled at a distance of 20 km from the enterprise was used as a model solution; the extract was prepared in accordance with [41] with a solid to liquid ratio (S: W) equal to 1: 5. The soil was mixed with distilled water for 3 min, then left for 5 min to settle. The organic carbon content in the extracts was about 40 mg/L, which is comparable to the average TOC content in the podzolic soils of the Kola Peninsula, according to the literature [42]. The experiments were carried out in a TCO-1/80 SPU thermostat at the selected temperatures (5 and 15 °C are the average monthly temperatures of the spring–autumn and summer periods).

In the model solutions, weighed portions of the dressing tailings of loparite ores (fraction < 0.071 mm) were added in the ratio S:L = 1:10. A series of similar experiments with distilled water was carried out as control experiments. The interaction time was 1, 3, 5 h with continuous stirring. The resulting solutions were filtered through a Vladipor membrane filter of the MFAS-OS-2 type (pore size 0.47 μm) and transferred for quantitative chemical analysis. The concentrations of elements in solutions were determined according to approved methods by ion exchange chromatography, atomic absorption spectrometry and inductively coupled plasma mass spectrometry.

The concentrations of elements in the resulting solutions were compared with the maximum permissible concentrations of harmful substances in the waters of water bodies of fishery significance (MPC_wbfs_), specified in the regulatory document of the Ministry of Agriculture of the Russian Federation [43]. A comparison with MPC_wbfs_—the limiting concentrations of pollutants that do not harm aquatic organisms—makes it possible to roughly judge the damage of the resulting solutions for aquatic ecosystems.

## 3. Results and Discussion

### 3.1. Study of Tail Material

**Figure 2:**
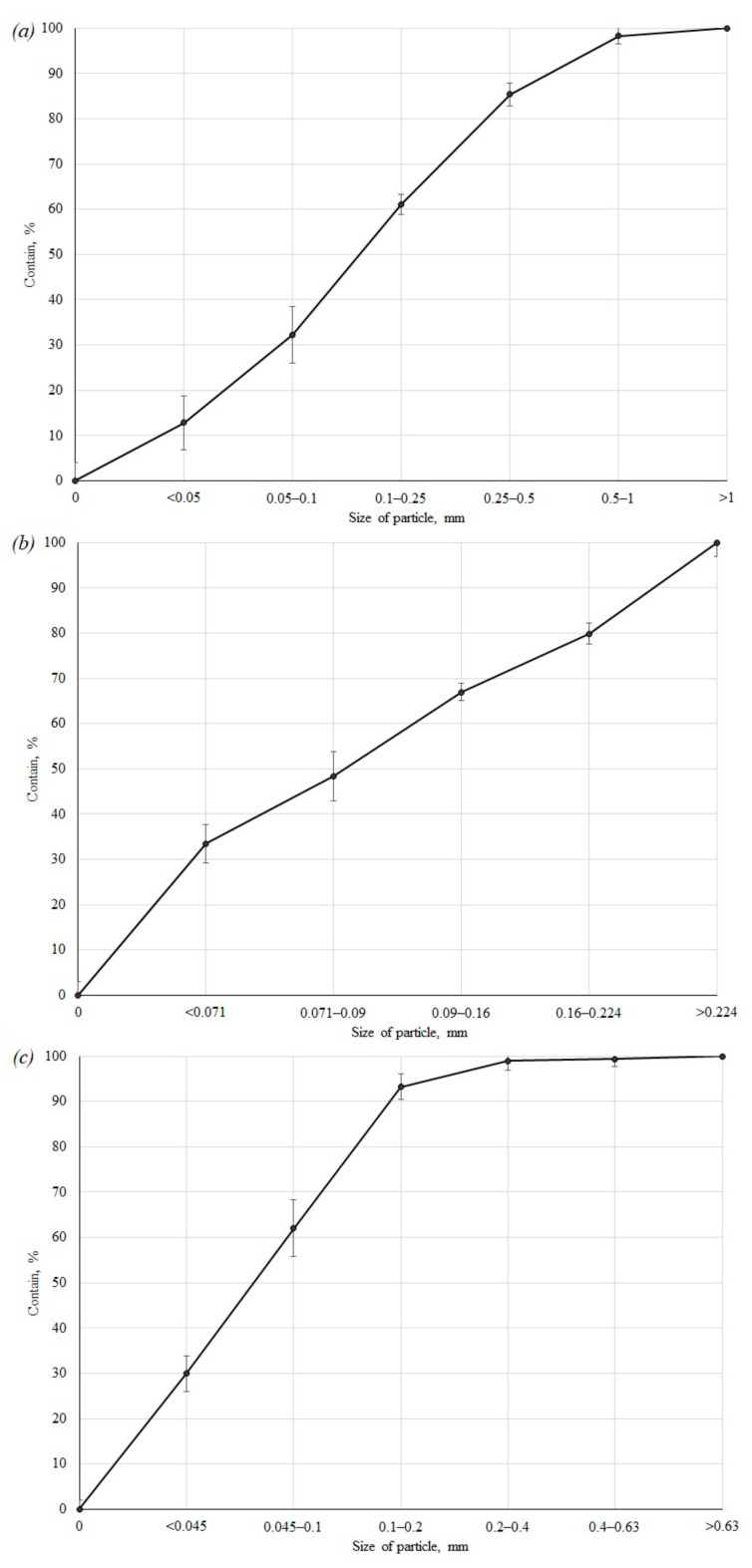
Cumulative curves of the granulometric composition: (**a**)—loparite ore dressing tails, (**b**)—apatite-nepheline ore dressing tails, (**c**)—complex ore dressing tails.

The cumulative curves of the granulometric composition of the investigated tailings material are shown in Figure 2.

Based on the results of the analysis of samples with enrichment, it can be concluded that they are classified as fine and medium-grained sands [44].

The predominant fractions for the trial concentration of loparite ores tailings are 0.25–0.5 mm and 0.1–0.25 mm; apatite-nepheline ores are <0.071 and 0.071–0.16 mm; complex ores are <0.045 and 0.045–0.1 mm. Due to wind erosion, when the surface of the tailing dump dries up in the summer, in dry, windy weather, there is a high probability of tailings dusting.

The value of the density of tailings sampled on the surface of the studied tailing dumps in natural occurrence varies in the range from 1.5 to 1.69 g/cm^3^, dry soil—from 1.42–1.74 g/cm^3^ and true density—2.6–3.0 g/cm^3^. A regular increase in the density of the material and tails with growth was noted (the correlation coefficient is quite high—0.6–0.8). The inverse correlation is typical for density and porosity, and the porosity coefficient is over 0.9. By the value of the coefficient of porosity ε—0.56–0.91—the samples are changed from loose soils to soils of medium density [45].

The results of determining the mineral and chemical composition are presented in Table 4 and Table 5.

The mineral composition is consistent with the results of silicate analysis. The predominant minerals composing the tailings of loparite ores are nepheline, feldspars and aegirine. Sodalite, loparite and apatite were found in impurity amounts. The mineral base of the tailings of apatite-nepheline ores is composed of nepheline, pyroxene and feldspars. Apatite, sphene, magnetite and calcite are present in smaller quantities. The mineral composition of the tailings of complex ores is quite different from the first two objects. The main minerals are forsterite, calcite and pyroxene. Smaller amounts were found: phlogopite, staffelite, feldspars, magnetite and apatite.

**Figure 3:**
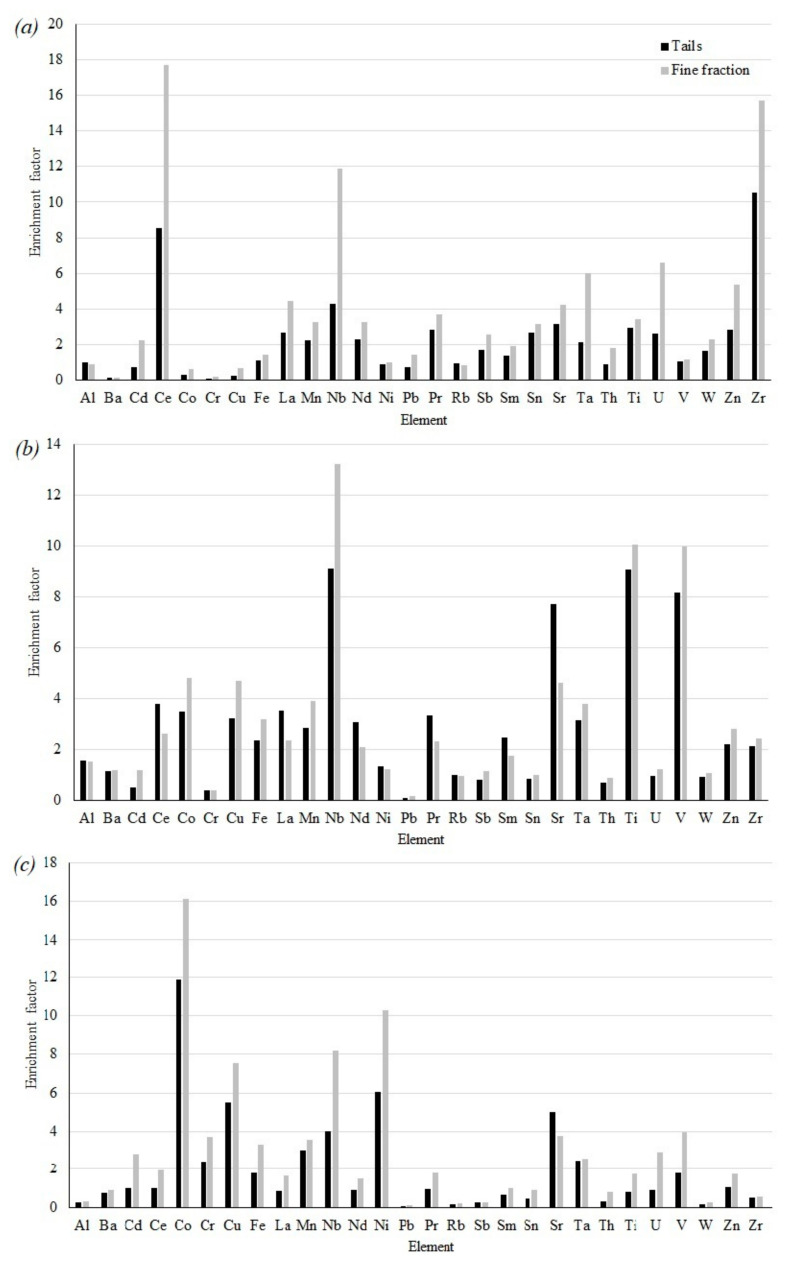
Enrichment factors: (**a**)—loparite ore dressing tails, (**b**)—apatite-nepheline ore dressing tails, (**c**)—complex ore dressing tails.

The calculated enrichment factors are shown in Figure 3. We studied both the averaged samples and the finely dispersed material (fine fraction) of the tailings (<0.071 mm).

It has been established that the tailings of the concentration of loparite ores are severe enrichment for Zr, moderately severe enrichment for Ce, moderate enrichment for Nb, Pr, Sr and Ti. Tailings of apatite-nepheline ore concentration are moderately severe enrichment for Nb, Sr, Ti and V, moderate enrichment for Co, Cu, Mn, Ta and LREE. Tailings of concentration of complex ores: severe enrichment for Co, moderately severe enrichment for Cu, Ni and Sr, moderate enrichment for Mn and Nb.

Attention is drawn to the fact that the finely dispersed material of the tailings is more enriched in microelements in comparison to the average sample in almost all cases. This is more pronounced for the tailings of the concentration of loparite ores: severe enrichment for Ce, Nb and Zr, moderately severe enrichment for Ta, U and Zn, moderate enrichment for La, Mn, Pr, Nd, Sr and Ti. Tailings of concentration of complex ores are severe enrichment for Co and Ni, moderately severe enrichment for Cu and Nb, moderate enrichment for Cr, Fe, Mn, Sr and V. The opposite tendency is noted for the finely dispersed material of tailings of apatite-nepheline ores severe enrichment for Nb, moderately severe enrichment Ti and V, moderate enrichment for Co, Cu, Mn, Sr and Ta.

Such an indicator as the index of geoaccumulation was also calculated for averaged samples and finely dispersed material of the investigated tailings. It was found that the tailings of loparite ore dressing are moderately polluted to highly polluted with Ce and moderately polluted with Sr. Tailings of apatite-nepheline ore dressing are moderately polluted to highly polluted with Sr and V and moderately polluted with Co, Cu and LREE. Complex ore dressing tailings are highly polluted with Co, moderately polluted to highly polluted with Ni, and moderately polluted with Cu, Mn and Sr.

Fine-dispersed material of loparite ore dressing tailings: highly polluted with Ce and moderately polluted with Mn, Sr, Zn, La, Pr and Nd. Tailings of apatite-nepheline ore dressing are moderately polluted to highly polluted with V and moderately polluted with Co, Cu, Mn and Sr. Complex ore dressing tailings are highly polluted with Co, moderately polluted to highly polluted with Cu and Ni and moderately polluted Cr, Mn, Sr and V.

In general, all three objects are characterized by high contents Mn and Sr, 1350–1805 ppm and 940–2300 ppm, respectively. In work [46], the sampling of soils and water bodies around Lang Mon apatite mine, mined by an open pit, and apatite mine disposal area near Tang Loong sorting plant IN Lao Cai Province, Viet Nam were carried out. The level of soil contamination by individual metals in the study area is given, while the average concentration of Zn was 7.07 mg/kg (in comparison with the results obtained in our study—131.3 mg/kg), Cu—6.88 mg/kg (in comparison—64.61 mg/kg), Cr—1.03 mg/kg (in comparison—9.802 mg/kg). Increased concentrations of Mn, Cu, Zn and other HMs from Yeshan iron mine tailings in the Jiangsu Province of China are also reported in the work [47].

The average content of light group REE in the tailings of the concentration of loparite and apatite-nepheline ores is 1150 and 770 mg/kg, respectively. These contents are quite significant, exceeding those found in the tailings of Ganzhou, Jiangxi province, in southern China [48]. Tang and coworkers (2016) reported that the total concentration of REE was 685 mg/kg, which is 3.4–3.9 times higher than the background concentration of 186.7 mg/kg in China [49].

The calculation results for the potential ecological risk index factor E_ir_ and the potential ecological risk index RI are shown in Table 6.

As can be seen, for most of the considered elements, the Potential ecological risk index factor E_ir_ in finely dispersed material is higher than for average samples. At the same time, the potential ecological risk index RI for finely dispersed material is 1.5–3 times higher than this parameter for average samples. It should be noted that the calculation was carried out only for heavy metals. It is highly likely that if rare earth metals were taken into account, the potential ecological risk index would be higher than low.

Previously, we carried out work to assess the impact of mining waste on the growth and development of higher plants [50]. Later, by the method of eluate phytotesting [51], an integral assessment of the toxicity of the finely dispersed material of the tailings and average samples of the tailings for the growth and development of the seed *Avéna satíva* L. has a toxic effect on the growth and development of higher plants. At the same time, no such effect was found for the average sample. According to the integral level of negative impact, the considered wastes were arranged in the following sequence: apatite-nepheline < complex < loparite [52].

As already known, solid mine wastes contain a significant amount of potentially toxic elements such as HM [53], which under suitable conditions may be released into the environment and subsequently change their geochemical characteristics [54,55].

### 3.2. Results of the Experiments

In Section 3.1, pollutants were identified that are characteristic for each of the objects under consideration.

#### 3.2.1. Mobilization of HM from Tailings of Concentration of Loparite, Apatite-Nepheline and Complex Ores

**Figure 4:**
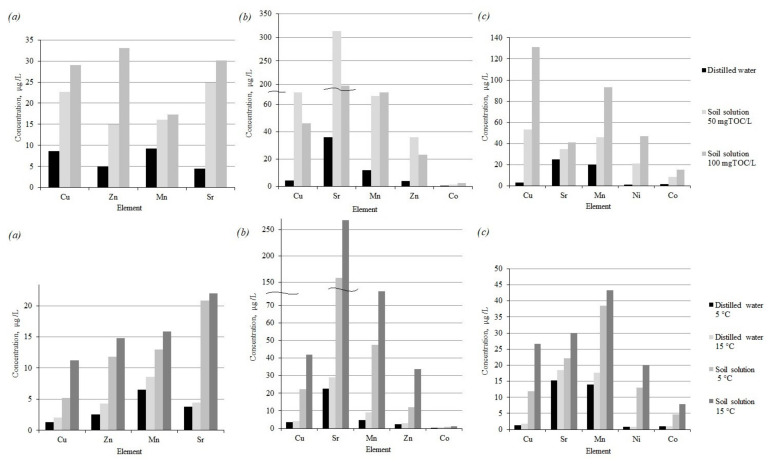
Concentrations of HM in the resulting solutions after five hours of interaction of enrichment tailings of loparite (**a**), apatite-nepheline (**b**) and complex (**c**) ores with distilled water and water extracts of soils.

Figure 4 shows the concentrations of HM after five hours of interaction between the finely dispersed material of the tailings and leaching agents: water extract of peat 50 and 100 mgTOC/L, with distilled water as a control; water extract of background soils 40 mgTOC/L, with distilled water as a control, at different temperatures, 5 and 15 °C, corresponding to the average temperatures of the spring–autumn and summer seasons.

The introduction of dissolved organic matter leads to the intensification of the transition of HM to the dissolved form. With an increase in the amount of organic matter, the HM concentration in the resulting solutions naturally increases. Additionally, when a decrease in the pH of solutions was noted, the stronger and more dissolved organic matter was introduced.

In the resulting solutions, after five hours of interaction between loparite ore dressing tailings and soil extracts, a significant excess of maximum permissible concentrations for fishery water bodies is observed for a number of elements: Cu exceeds MPC_wbfs_ (0.001 mg/L) by 23–29 times, Zn—MPC_wbfs_ = 0.01 mg/L—by 1.5–3.3 times and Mn by 1.6–1.7 times (MPC_wbfs_ = 0.01 mg/L) at the content of 50 and 100 mgTOC/L in the soil extract, respectively. Sr actively transforms into a dissolved form, but the maximum permissible concentration does not exceed.

In the resulting solutions, after five hours of interaction between apatite-nepheline ore dressing tailings and soil extracts, there is a significant excess of the maximum permissible concentrations for fishery water bodies for a number of elements: Cu exceeds MPC_wbfs_ (0.001 mg/L) by 42–68 times, Mn by 6.4–6.7 times (MPC_wbfs_ = 0.01 mg/L) and Zn—MPC_wbfs_ = 0.01 mg/L—by 2.2–3.6 times, with the content of 50 and 100 mgTOC/L in the soil extract, respectively.

In the resulting solutions, after five hours of interaction between the tailings of complex ores and soil extracts, there is a significant excess of the maximum permissible concentrations for fishery water bodies for a number of elements: Cu exceeds the MPC_wbfs_ (0.001 mg/L) by 52–129 times, Mn by 4.3–9.1 times (MPC_wbfs_ = 0.01 mg/L), Ni—MPC_wbfs_ = 0.01 mg/L—by 2–4.4 times and Co—MPC_wbfs_ = 0.01 mg/L—by 1.1–1.6 times when the soil extract contains 50 and 100 mgTOC/L, respectively.

An increase in temperature in the second series of experiments also intensifies the process of HM mobilization from the finely dispersed material of the tailings.

In the article [56], the addition of humic substances that can accelerate the migration of Cu into the surrounding environment was investigated. The influence of dissolved organic matter on more intensive leaching of Cu and Ni from contaminated bottom sediments was studied in [57]. A number of works are devoted to the assessment of the use of compost and additives containing organic matter in contaminated soils. Cases of both mobilization and immobilization of HM were noted [58,59,60].

Note that a strong mobilization ability of organic acids that are part of the soil water extract in relation to trace elements was previously noted for Zn, Cu, Mn, Th, V, La, Nb, etc., in [61].

High Cu content in soils can have a chronic toxic effect on the environment and living organisms [62] due to its high persistence and bioaccumulation potential [63].

The distribution of Zn in soils is determined by a complex of precipitation, complexation, and adsorption reactions. An excess of Zn causes serious consequences in plants [64], such as a decrease in yield and growth retardation, chlorosis caused by iron deficiency due to a decrease in chlorophyll synthesis and degradation of chloroplasts, as well as impaired absorption of phosphorus, Mg and Mn [65,66].

The toxicity of excess Mn in plants leads to chlorosis of young leaves and necrosis of mature leaves, which makes it difficult for plants to grow [67,68,69]. Symptoms of Mn toxicity vary widely among plant species and varieties.

When Ni enters the soil, it causes high oxidative stress in the systems of mammals and terrestrial plants and leads to disruption of ionic homeostasis [70]. Ingestion of Ni-contaminated food crops can contribute to liver damage and stomach problems, as well as neurological complications [71].

The presence of Co in the soil in significant amounts poses a significant threat to plant growth and development [72]. Co is highly mobile and readily adsorbed with toxic effects, including leaf chlorosis, plant growth retardation and photosynthetic rate [73]. The article [74] noted that cobalt is very toxic in high concentrations. In studies on test systems in mammals, high concentrations of Co ions had cytotoxicity and caused apoptosis, with an increase in concentration and necrosis with an inflammatory reaction.

#### 3.2.2. Mobilization of REE from Tailings of Concentration of Loparite, Apatite-Nepheline and Complex Ores

**Figure 5:**
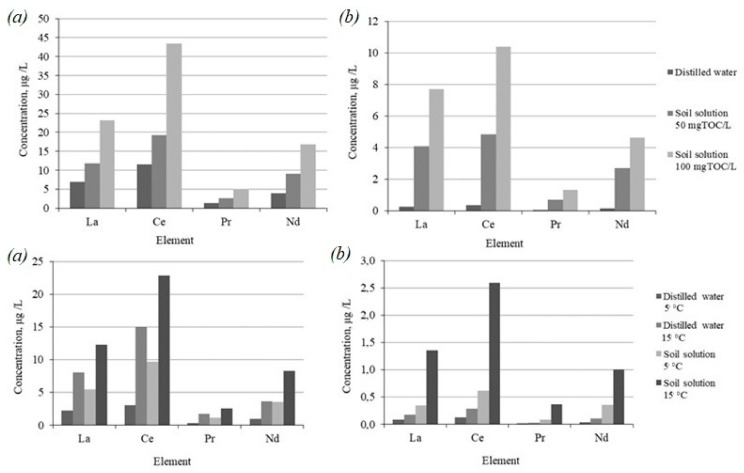
Concentrations of REE in the resulting solutions after five hours of interaction of enrichment tailings of loparite (**a**) apatite-nepheline (**b**) with distilled water and water extracts of soils.

Figure 5 shows the concentrations of REE after five h of interaction between the finely dispersed material of the tailings and leaching agents: water extract of peat 50 and 100 mgTOC/L, distilled water as a control; water extract of background soils 40 mgTOC/L, with distilled water as a control, at different temperatures, 5 and 15 °C, corresponding to the average temperatures of the spring–autumn and summer seasons.

The introduction of organic matter leads to a sharp acceleration of the process of the transition of REE into the solution. Similar results were obtained in [75,76], where the studied materials—tailings, ore containing REE—were leached with separate organic acids. The total increase in the mass of dissolved REE is proportional to the increase in the introduced organic matter. The process is more intensive for lanthanum, cerium, neodymium for the tailings of loparite ore dressing. As in the case of HM, an increase in temperature and the addition of dissolved organic matter naturally intensifies the process of the transition of REE into soluble forms.

Substances passing into the aqueous extract represent the most mobile part of organic matter. Organic acids cause the decomposition of primary minerals, especially during weathering of rocks rich in mineralogical composition and participate in the migration of mineral destruction products along the soil profile [77].

The migration of REE in soils is determined by the redox potential of soils, pH level and adsorption/desorption reactions associated with organic matter and Fe and Mn oxides, soil texture [78,79,80].

The stability of the bond of REE with humic substances is different; therefore, under the influence of dissolved organic matter, the reverse process, desorption, can occur [81]. The REE concentration in the soil solution has a direct correlation with the content of dissolved organic carbon and an inverse correlation with the soil pH [82,83]. In general, the behavior of REE in soils is similar to that of heavy metals [18]. MPCs in water bodies and soils for these elements have not been established, the effect of REEs on biota has not been sufficiently studied.

The toxicity of REEs is closely related to the closeness of their physicochemical properties. REEs can enter plants both through the root system and upon deposition on their aboveground parts. In a study [84], a change in the absorption of REE by plants under their joint action was noted. For example, the combination of Ce with La increases the uptake of Ce, and Nd in combination with La, Ce and Pr increases the uptake of Nd by plants while reducing the uptake of Ce. In living organisms, REEs can form chelates with substances involved in metabolism, for example, with proteins, nucleic acids, amino acids, pigments, etc. [85]. Many studies have noted the ability of REEs to replace calcium ions in organisms [86,87], including in enzymes, thereby interfering with the normal physiological course of enzymatic reactions.

In recent years, more and more works have been published describing the toxicity of REEs [88,89,90]; however, standards that ensure environmental protection have not yet been established.

### 3.3. Condition of Environmental Components in the Zone of Influence of Tailings

Comprehensive assessments of the state of environmental components in the zone of influence of ore dressing waste storage facilities in the study area have not previously been carried out. There are scattered reports on studies of bottom sediments of lakes and water bodies.

Thus, the analysis of bottom sediments of Lake Kovdor, located in the impact zone of the tailing dump of complex ores, showed significant values of the HM pollution coefficient—from 2.3 to 18.0 [91]. According to the classification of L. Hakanson (1980), they refer to significant and high pollution. The highest Cf values are observed for Co, Ni, Cu, metals that are toxic and hazardous in high concentrations for aquatic organisms, which are part of the tailings of complex ores. The bottom sediments of a lake located near the apatite-nepheline ores tailing dump were investigated in [92]. Since the maximum permissible REE content in bottom sediments has not been established, the study compared the content with the maximum for freshwater ecosystems and the content in Lake Baikal. Abnormally high contents of not only HM but also REEs were found in the tailings [92].

More data was obtained when conducting a geoecological assessment of environmental components in the zone of influence of the tailing dump of loparite ores in the framework of the dissertation research of the author of the article. In the course of the study, halos of dust pollution of soils and bottom sediments of Lake Ilma, located next to the tailing dump, coinciding with the wind rose, were established. The bottom sediments contain anomalously high contents of HM and REE, exceeding even the contents in Lake Imandra, which is subject to contamination during the processing of apatite-nepheline ores [93]. Soil and plant samples taken in the impact zone were also characterized by increased contents of Mn, Sr, Zn and LREE, which are part of the tailings of loparite ore dressing [94].

Based on the results of the assessment of long-term trends in the temperature and humidity of the territory of the Kola Peninsula, scientists from the Federal Research Center of the Kola Science Center of the Russian Academy of Sciences made a conclusion about continuing warming and an increase in precipitation [95], which can change such soil properties as pH and organic matter content [20,21], which is likely to further affect the availability of HM.

## 4. Conclusions

According to the results of studying the tailings of apatite-nepheline, complex and rare earth ores, the material was assigned to fine and medium-grained sands; in terms of the porosity coefficient, the samples vary from loose soils to soils of medium density. All studied materials are characterized by the presence of a fraction of less than 0.071 mm, which indicates the likelihood of dusting the tailing dumps during the snowless period. The finely dispersed material of the tailings is, in almost all cases, more enriched in microelements than the average sample. The fine fraction of loparite ores in comparison with the average sample is significantly enriched in Zr and Ce, moderately in Nb, Pr, Sr and Ti. Tails of apatite-nepheline ores are moderately strongly enriched in Nb, Sr, Ti and V and moderately enriched in Co, Cu, Mn, Ta and LREE. Complex ore tailings: highly enriched in Co, moderately highly enriched in Cu, Ni and Sr and moderately enriched in Mn and Nb.

The calculated geoaccumulation indexes for the average sample and fine fraction showed a moderate level of contamination of the tailings of loparite ores with Ce and Sr and of apatite-nepheline Sr, V, Co, Cu and light REE. For complex ores, heavy contamination of Co, from moderate to strong Ni, moderate to Cu, Mn, Sr was determined.

The index of potential environmental risk shows a level from low to medium (for complex ores), while for most of the considered elements, the level of potential risk for fine fractions is 1.5–3 times higher than for the average sample. It should be noted separately that this index was calculated for HM. This conclusion is in good agreement with the carried out eluate phytotesting of the studied materials (on the test object *Avéna satíva* L.). In terms of the integral level of negative impact, the highest indicator is for loparite ores, followed by complex and apatite-nepheline ores.

Experiments on the mobilization of environmentally hazardous elements from the tailings material at different temperatures and interaction with the organic matter have been carried out. As a source of water-soluble organic matter, we used soil extracts with a TOC content of 50 and 100 mg/L. The temperature regime was chosen in accordance with the average temperatures of spring and summer on the Kola Peninsula. It was found that the transition of environmentally hazardous elements—both HM and REE—into the dissolved form enhances the temperature rise, the increase in the introduced organic matter and the decrease in the pH of the solutions.

The results of this work should be taken into account when carrying out reclamation work since the biological stage is characterized by an increase in the content of organic matter and an increase in soil acidity, which can lead to a change in the migration ability of pollutants.

## Figures and Tables

**Table 1:** Clark content for some elements by [31], mg/kg.

Al	Ba	Cd	Ce	Co	Cr	Cu	Fe	La	Mn	Nb	Nd	Ni	Pb
77,000	830	0.10	100	5	25	20	27,000	60	600	20	46	8	20
Pr	Rb	Sb	Sm	Sn	Sr	Ta	Th	Ti	U	V	W	Zn	Zr
12	200	0.26	9	3	300	3.5	18	2300	3.5	40	1.5	60	200

**Table 2:** The enrichment factor EF_𝑖_ and geoaccumulation index I_geo_.

EF_i_ Value	Enrichment Level	I_geo_ Value	I_geo_ Class	Pollution Level
<1	no enrichment	≤0	0	unpolluted
1–3	minor enrichment	0–1	1	unpolluted to moderately polluted
3–5	moderate enrichment	1–2	2	moderately polluted
5–10	moderately severe enrichment	2–3	3	moderately polluted to highly polluted
10–25	severe enrichment	3–4	4	highly polluted
25–50	very severe enrichment	4–5	5	highly polluted to very highly polluted
>50	extremely severe enrichment	>5	6	very highly polluted

**Table 3:** The potential ecological risk index factor Eir and potential ecological risk index RI.

Er Value	Ecological Risk	RI Value	Ecological Risk
<40	low ecological risk	<150	low ecological risk
40–80	moderate ecological risk	150–300	moderate ecological risk
80–160	appreciable ecological risk	300–600	high ecological risk
160–320	high ecological risk	>600	significantly high ecological risk
>320	serious ecological risk		

**Table 4:** The mineral composition of the ore dressing tails, %.

	Loparite Ore Dressing Tails	Apatite-Nepheline Ore Dressing Tails	Complex Ore Dressing Tails
Nepheline (Na,K)AlSiO_4_	59.57	59.51	-
Feldspars (Na,K)AlSi_3_O_8_	16.07	11.10	1.67
Apatite Ca_10_(PO_4_)_6_(OH,F,Cl)_2_	1.05	5.55	0.24
Loparite (Na,Ce,Ca,Sr,Th)(Ti,Nb,Fe)O_3_	0.94	-	-
Aegirine NaFe(Si_2_O_6_)	20.42	-	-
Sodalite 3Na₂O·3Al₂O₃·6SiO₂·2NaCl	1.58	-	-
Pyroxene SiO_4_	-	17.90	18.58
Sphene CaTi(SiO_4_)O	-	3.61	-
Magnetite FeO·Fe_2_O_3_	-	2.26	1.50
Calcite CaCO_3_	-	0.37	29.00
Forsterite Mg_2_(SiO_4_)	-	-	32.35
Staffelite	-	-	6.15
Phlogopite KMg_3_(Si_3_AlO_10_)·(F,OH)	-	-	9.72

**Table 5:** The chemical composition of the ore dressing tails, weight %.

	SiO_2_	TiO_2_	Al_2_O_3_	Fe_2_O_3_	FeO	MnO	CaO	MgO	K_2_O	Na_2_O	P_2_O_5_	SrO	F	SO_3_	LoI *	∑
Loparite ore dressing tails	48.08	1.1	22.47	5.3	0.66	0.23	1.63	0.45	4.24	13.33	0.79	0.33	0.08	0.08	1.2	99.97
Apatite-nepheline ore dressing tails	42.56	2.65	21.35	4	2.27	0.16	5.78	1.31	6.09	9.4	2.3	0.27	0.25	0.14	1.32	99.85
Complex ore dressing tails	25.46	0.37	3.42	1.81	4.16	0.2	24.07	19.45	1.27	1.32	2.6	0.24	0.13	1.21	14.9	100.66

* loss on ignition.

**Table 6:** The potential ecological risk index factor Eir and the potential ecological risk index RI.

Object	Potential Ecological Risk Factor Er								RI	Risk Grade
	Cd	Cr	Cu	Mn	Ni	Pb	Zn	Co		
Apatite-nepheline ore dressing tails	14.56	0.78	16.15	2.85	6.72	0.40	2.19	6.99	50.65	low
Apatite-nepheline ore dressing tails, fine fraction	35.67	0.72	23.49	3.91	6.04	0.70	2.80	9.61	82.94	low
Complex ore dressing tails	30.29	4.86	27.56	3.01	30.33	0.36	1.04	23.71	121.15	low
Complex ore dressing tails, fine fraction	85.49	7.44	37.68	3.59	51.46	0.50	1.71	32.24	220.11	moderate
Loparite ore dressing tails	21.55	0.16	1.34	2.25	4.63	3.71	2.86	0.60	37.10	low
Loparite ore dressing tails, fine fraction	67.79	0.40	3.33	3.26	4.98	7.19	5.35	1.24	93.54	low

## Data Availability

Not applicable.
